# Mitochondrial DNA Modification in Assisted Reproduction: Concept to Practice—A Narrative Review

**DOI:** 10.3390/ijms27062890

**Published:** 2026-03-23

**Authors:** Mariam Mehwish Mohsin, Misbah Azher, Fatima Asghar, Hiba Habeebu Rahiman, Rajani Dube, Subhranshu Sekhar Kar, Shadha Nasser Mohammed Bahutair, Bellary Kuruba Manjunatha Goud, Swayam Siddha Kar

**Affiliations:** 1 Department of Obstetrics and Gynecology, RAK College of Medical Sciences, RAKMHSU, Ras Al-Khaimah P.O. Box 11172, United Arab Emirates; maryammehwish@gmail.com (M.M.M.); misbahazher@gmail.com (M.A.); hibarahiman99@gmail.com (H.H.R.); shadha@rakmhsu.ac.ae (S.N.M.B.); 2 Department of Internal Medicine, RAK College of Medical Sciences, RAKMHSU, Ras Al-Khaimah P.O. Box 11172, United Arab Emirates; fatimaasgharr3000@gmail.com; 3 Department of Paediatrics and Neonatology, RAK College of Medical Sciences, RAKMHSU, Ras Al-Khaimah P.O. Box 11172, United Arab Emirates; subhranshu.kar@rakmhsu.ac.ae; 4 Department of Biochemistry, RAK College of Medical Sciences, RAKMHSU, Ras Al-Khaimah P.O. Box 11172, United Arab Emirates; manjunatha@rakmhsu.ac.ae; 5 Department of Obstetrics and Gynecology, School of Medicine, University of Lancashire, Preston Campus, Preston PR1 7BH, UK; swaekar35@gmail.com

**Keywords:** mitochondria, mitochondrial diseases, DNA modification, mitochondrial replacement therapy, assisted reproductive technique

## Abstract

Mitochondria play a fundamental role in human reproduction by supplying the energy required for key early reproductive processes. As mitochondrial Deoxyribonucleic acid (mtDNA) is maternally inherited, pathogenic mutations can lead to multisystem disorders that are transmitted to offspring. Mitochondrial replacement therapy (MRT) has emerged as a promising assisted reproductive approach to prevent the transmission of pathogenic mtDNA by replacing defective mitochondria with healthy donor mitochondria. There have been recent reports of successful MRT in humans. However, MRT remains a relatively new procedure and needs further experiments to establish its long-term safety and effectiveness. Overall, mitochondrial replacement therapy holds significant promise in helping families build healthier futures. This review explores the evolution of mitochondrial DNA modification in reproductive cells and addresses the associated ethical considerations, including acceptable clinical indications, reproductive choices, and long-term considerations for affected children.

## 1. Introduction

Mitochondria are double-membrane-bound organelles essential for energy production in human cells. They generate adenosine triphosphate (ATP) through oxidative phosphorylation, thereby supporting cellular metabolism and physiological functions. Beyond energy generation, mitochondria regulate apoptosis, calcium homeostasis, heat production, and the synthesis of key metabolites [1]. They contain their own circular DNA. Mitochondrial DNA (mtDNA) is made up of 37 genes, making up <0.1% of our body’s total DNA. Many human diseases have been attributed to dysfunctions due to gene mutations [2,3,4,5,6]. Mitochondrial dysfunction is reported to result from mutations in the nuclear DNA (nDNA) or mtDNA sequences inherited through the oocyte. Mitochondrial dysfunction is implicated in a wide range of human diseases, including neurodegenerative disorders, metabolic syndromes, and inherited mitochondrial disorders, underscoring their central role in cellular health and survival [7].

As only maternal mitochondrial DNA material is transmitted to the offspring during fertilization, diseases caused by mtDNA mutations follow a maternal inheritance pattern. While both sexes can inherit the disease, only women are at risk of passing it on to their children [8]. This results in a dominant transmission and manifestation of different diseases [9]. In such patients, MRT provides a promising therapeutic approach for the individual [10]. MRT is a modified assisted reproductive technique designed to prevent transmission of diseases caused by pathogenic mitochondrial DNA mutations from mother to child.

### Rationale for Mitochondrial Replacement Therapy and Its Clinical Applications

The procedure involves several vital steps. First, oocytes are obtained from both the affected mother and a healthy donor through controlled ovarian stimulation. The nuclear genetic material from the mother’s oocyte, which carries the parental chromosomes, is then carefully removed and transferred into a donor oocyte that retains healthy mitochondria. The reconstructed oocyte is subsequently fertilized with sperm from the intended father using the intracytoplasmic sperm injection (ICSI) method. The resulting embryo is cultured in vitro until it reaches the blastocyst stage and is then transferred to the uterus of the intended mother [11,12]. MRT has been widely recognized as a therapeutic approach to prevent the transmission of several mitochondrial disorders such as Leigh syndrome, mitochondrial encephalomyopathy with lactic acidosis and stroke-like episodes (MELAS), myoclonic epilepsy with ragged-red fibers (MERRF), and Leber’s hereditary optic neuropathy (LHON), in which the primary cause involves pathogenic mtDNA mutations affecting cellular energy metabolism [7,12]. Beyond disease prevention, MRT has also been explored in assisted reproductive technology for women with poor oocyte quality, age-related mitochondrial decline, or repeated implantation failure. By improving mitochondrial function within oocytes, this approach may enhance oocyte quality and contribute to improved reproductive outcomes [13,14]. These potential benefits have generated considerable interest in MRT as a promising therapy in reproductive medicine; however, several scientific, ethical, and regulatory aspects still require further investigation [15,16].

However, a recent study raised concerns that even a small presence of abnormal mitochondria during nuclear transfer can proliferate in the oocyte, with a stochastic bottleneck leading to genetic drift. For MRT to be used as a reliable method for preventing mitochondrial diseases, researchers need to find a way to counteract this genetic drift [9]. The techniques designed for MRT raise numerous ethical, social, and policy issues due to their controversial method of using the cytoplasm from a donated egg containing the genetic material in the form of mtDNA. Nonetheless, if effective, it could satisfy the desire of women with maternally inherited mitochondrial disease to have a healthy, genetically related child but with a substantially reduced risk of passing on mtDNA disease [10].

## 2. Result and Discussion

### 2.1. Theoretical Framework of MRT

Role of Mitochondria in Gametes and Embryos

**Figure 1:**
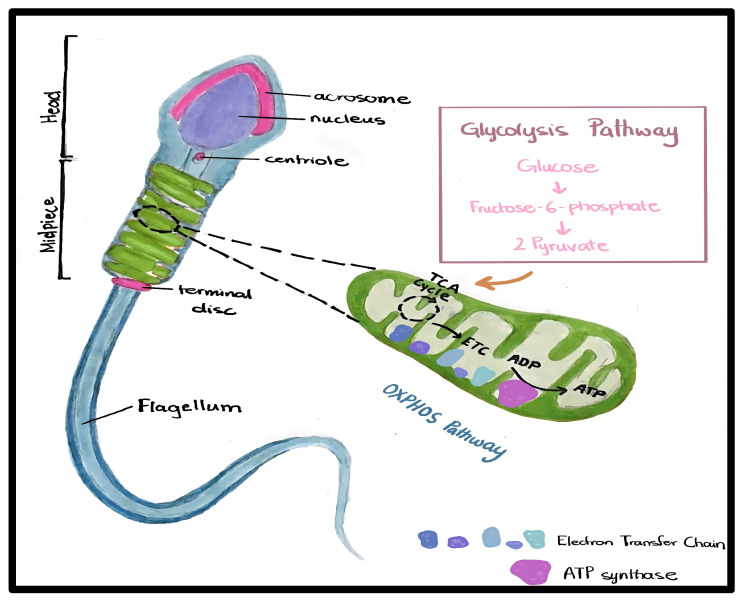
Mitochondria location and function in mature sperm. Mitochondria are tightly packed in a spiral arrangement in the midpiece of the sperm. They produce ATP through two pathways: glycolysis and OXPHOS. This energy supports crucial sperm functions, including flagellar movement and the acrosome reaction needed for fertilization, as shown in glycolysis and the OXPHOS pathway.

Mitochondria play an important role in the facilitation of sperm movement, regulating calcium levels, and controlling apoptotic pathways [17] [Figure 1].

**Figure 2:**
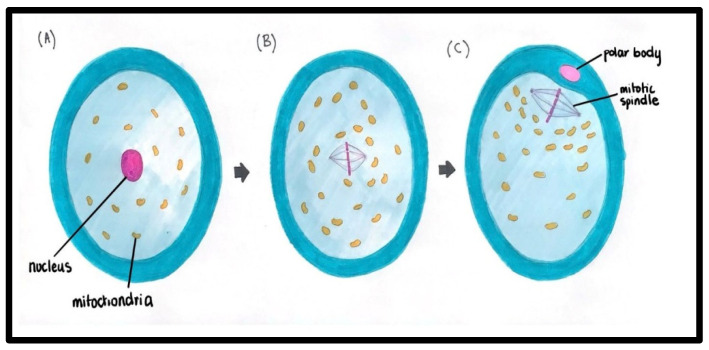
Redistribution of mitochondria in maturing oocytes. (**A**) Germinal vesicle (GV): Mitochondria are evenly distributed throughout the ooplasm. (**B**) Metaphase I (MI): Mitochondria aggregate to form clusters while maintaining a relatively homogeneous distribution across the ooplasm; these clusters are predominantly localized toward the inner cytoplasmic region. (**C**) Metaphase II (MII): Mitochondria show further aggregation, forming more pronounced clusters that are increasingly relocated toward the inner ooplasm. Mitochondria, nucleus, polar body, and mitotic spindle are shown in the figure.

These are important for the fusion of sperm with oocytes [10]. Mitochondria are also redistributed in mature oocytes during gametogenesis [Figure 2].

Moreover, mtDNA is more susceptible to cumulative oxidative damage compared to nDNA due to its proximity to sites of mitochondrial oxidative metabolism and the absence of DNA-associated proteins that shield nuclear DNA from oxidative damage [18].

### 2.2. Techniques for Mitochondrial Replacement Therapy

Mitochondrial replacement therapy is a new form of IVF that has recently emerged as a strategy to prevent the transmission of severe mitochondrial diseases [14,15,16]. There are several processes by which MRT can be performed: pronuclear transfer (PNT), spindle fiber transfer (ST), and polar body transfer (PBT). PNT and MST processes use a donor egg that has normal mtDNA [12,19,20].

#### 2.2.1. Pronuclei Transfer (PNT)

**Figure 3:**
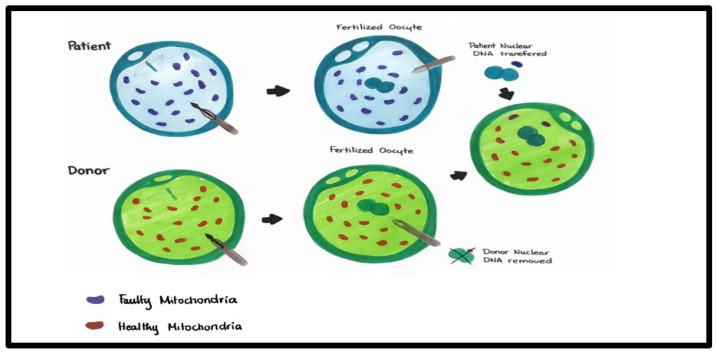
Pronuclear transfer (PNT). PNT is a technique where the pronuclear DNA from a fertilized oocyte with abnormal mitochondria is transferred to a donor zygote, with its pronuclear DNA discarded. The process has been demonstrated in the figure.

In PNT, the pronuclei from the intended parents’ zygote are transferred into an enucleated donor zygote, resulting in reconstructed embryos with minimal mtDNA carryover that can develop to the blastocyst stage. Pronuclei can be transferred between fertilized human zygotes with limited mtDNA carryover (<2%). Consequently, the reconstructed zygote is cultured in vitro until it reaches the blastocyst stage, where it undergoes genetic testing to assess levels of mtDNA heteroplasmy, detect chromosome abnormalities, and determine sex, if applicable [Figure 3].

Embryos meeting the predetermined criteria for these parameters are then transferred into the uterus of the intended mother for pregnancy [10]. In summary, metaphase II (MII) transfer and PN transfer are novel approaches with potential for preventing mtDNA diseases, subject to addressing all safety and efficacy protocols. However, neither method ensures total eradication of patient mtDNA [21]. A study led by Douglass Turnbull of the Newcastle group had initially executed experiments using fertilized zygotes, which are usually disposed of during ARTs. It was discovered that reconstructed zygotes exhibit about 50% of developmental potential in comparison to unmanipulated fertilized control zygotes. Procedure enhancement techniques led the group to successfully minimize mtDNA carryover, with levels ranging from undetectable to 11.4 percent. They also noted that in some cases, mtDNA carryover levels were undetectable or <2% [10].

#### 2.2.2. Spindle Transfer (ST)

**Figure 4:**
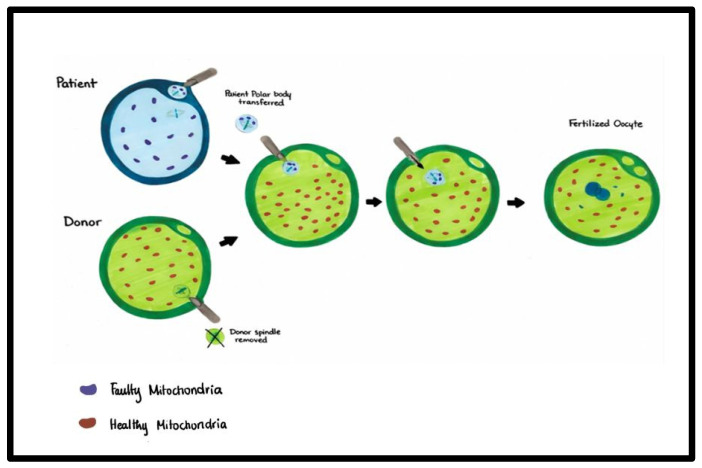
Maternal spindle transfer (MST). MST involves transferring the nuclear genetic material of a patient’s oocyte with defective mitochondria to a donor oocyte with its nuclear material removed. The process of MST has been demonstrated here.

This methodology involves removing chromosome–spindle complexes from the oocyte with mutated mtDNA to be transferred into the donor oocyte. MII conveyance is less invasive than germinal vesicle transfer (GV), as the chromosomes can be easily aspirated into small amounts of mitochondrial transfer [Figure 4].

While there have been reports of successful live births after ST, some studies showed less favorable outcomes, leading to about half of the manipulated oocytes failing to fertilize. The increased rates of failure were highly associated with premature oocyte activation, proving that human MII oocytes are quite sensitive to spindle manipulation [21]. The oocyte of the intended mother with mutated mtDNA will be discarded. Instead, the nuclear chromosomes of the intended mother will be inserted into the provided oocyte containing non-pathogenic mtDNA. This oocyte would then be fertilized with the sperm of the intended father or another male donor. The resulting embryo would undergo diagnostic testing, including preimplantation genetic diagnosis (PGD), to ensure its viability. Once the embryos are deemed suitable for transfer based on test results, they would be frozen until they are ready for transfer into the uterus of the intended mother [10].

A study conducted by Paull et al. (2013) [22] at the New York Stem Cell Foundation demonstrated the practicality of MST in human oocytes. Metaphase II oocytes were parthenogenetically activated to prevent the creation and disposal of potentially viable embryos. After MST and artificial activation, an average of 0.36 percent mtDNA carryover was detected in MST zygotes [10]. Clinics from the US and Spain have effectively implemented the chromosome–spindle transfer technique, despite lacking official approval from local authorities. In one instance, the technique was utilized to prevent maternal transmission of the known mtDNA mutation 8993T>G, associated with Leigh syndrome. This treatment involved transferring the patient’s metaphase chromosomes into donor oocytes that had been previously enucleated. The procedure took place in Mexico and led to the birth of a healthy baby boy [23].

#### 2.2.3. Polar Body Transfer (PBT)

**Figure 5:**
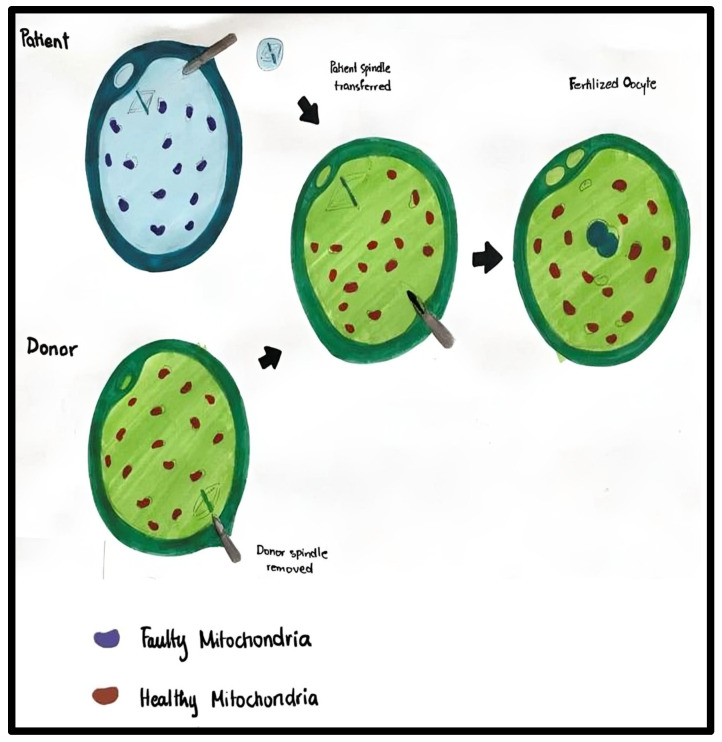
Polar body transfer (PBT). PBT is a technique where the polar body of the patient is transferred to the donor oocyte. The nucleus of the donor oocyte containing the genetic material is removed and replaced by the genetic material of the patient contained in the polar body. The process of PBT has been demonstrated here.

Polar body 1 transfer (PB1T) and polar body 2 transfer (PB2T) have been recently recognized as possible alternative or supplementary approaches to prevent the transmission of mtDNA diseases. PB1T involves transferring the first polar body, while PB2T involves transferring the second polar body to either an enucleated or partially enucleated mature oocyte or zygote [Figure 5].

**Figure 6:**
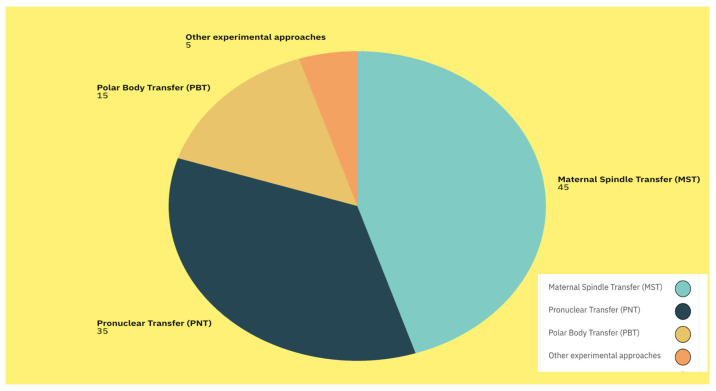
Distribution of mitochondrial replacement therapy techniques reported in the literature: MST—45%; PNT—35%; PBT—15%; and other approaches—5%.

These advantages may include reduced levels of mtDNA carryover, avoidance of cytoskeletal inhibitors, and less invasive manipulation procedures. According to a review conducted by the Human Fertilization and Embryology Authority (HFEA), although these techniques are still in their early stages of development, as potential MRT, PB1T, and PB2T could offer potential advantages over MST and PNT [8]. Wang et al. [24] demonstrated that substituting polar body 2 (PB2) for the maternal pronucleus in mice resulted in the development of reconstructed zygotes to the blastocyst stage and the birth of normal offspring. Later, replacing the female pronucleus with PB2T as soon as the pronuclei became visible led to poor developmental potential of PB2T-derived embryos in humans. Further investigations are necessary to address this issue [23]. Available literature reports MST as the most common type of MRT used [Figure 6].

Additionally, there are emerging and more controversial technologies, such as gene editing techniques utilizing CRISPR/Cas9 [21]. Mitochondrial genome editing, while not yet accessible for clinical application, is currently being investigated. These therapies aim to overcome the need for third-party participation (oocyte donors) and alleviate discomfort linked with patient oogonia stem cell retrieval. It is crucial to adapt existing genome editing techniques, proven effective for nuclear DNA, to effectively target mtDNA. CRISPR/Cas9 genome editing technology was first demonstrated in 2012 and has emerged as a groundbreaking tool for nuclear gene editing. Recent research indicates that Cas9 can be easily programmed by a guide RNA (gRNA) to target various cellular sites. Consequently, significant efforts have been directed toward refining this system to eradicate mutant mtDNA [18].

### 2.3. Autologous Mitochondrial Enhancement Techniques in ART

#### 2.3.1. Autologous Mitochondrial Transfer (AUGMENT^®^)

Autologous mitochondrial transfer has been developed in response to concerns about mitochondrial heteroplasmy associated with heterologous mitochondrial enrichment. The procedure known as AUGMENT^®^ involves isolating mitochondria from a patient’s own egg-precursor cells and injecting them into her oocytes at the time of ICSI, together with spermatozoa [25]. Since 2014, this technique has been tested in 166 women involved in three different studies performed by the UAE, Canada, and Spain. Fakih and colleagues reported the application of the AUGMENT technique in 59 patients from the Fakih IVF clinic in the UAE and 34 patients from TCART Fertility Partners in Canada. In this study, pregnancy rates exceeded the patients’ previous IVF outcomes, with ongoing pregnancies increasing approximately 11-fold at the Fakih IVF Clinic in the UAE and 18-fold at TCART Fertility Partners in Canada [26]. Clinical investigations of autologous mitochondrial transfer (AUGMENT^®^) have also reported improved reproductive outcomes in selected patient populations. In one multicenter clinical study, it was reported that, in the UAE center, the clinical pregnancy rate increased from 4% in previous IVF cycles to 22% after AUGMENT, while in the Canadian center, pregnancy rates improved from 11% to 35% following treatment [26].

Despite differences in clinic protocols and study settings, a key strength of this research was the intrapatient, intracycle design: within the same ovarian stimulation cycle, retrieved oocytes were randomized 1:1 to undergo either standard ICSI or the AUGMENT protocol, minimizing potential bias [27]. However, a randomized controlled trial conducted in Spain with 59 patients who were reported to show no significant improvement, with ongoing pregnancy and live birth rates of 41.2% in the AUGMENT group versus 41.7% in controls. These mixed findings highlight the need for larger randomized trials to determine the clinical effectiveness of autologous mitochondrial transfer [28,29].

#### 2.3.2. Stem Cell–Derived Mitochondrial Transfer

A newer autologous method uses mitochondria from a patient’s own stem cells for oocyte rejuvenation. This approach helps bypass donor cytoplasm and aims to restore mitochondrial function. Several studies highlight the potential of mitochondria harvested from MSCs, induced pluripotent stem (iPS) cells, adipose-derived stem cells, urine-derived MSCs, and other adult stem-cell sources [29]. Animal studies have shown that stem-cell mitochondria are usually healthier, produce more ATP, and generate fewer reactive oxygen species. When these mitochondria were injected into oocytes from older mice, improvements were observed in fertilization rates, embryo development, and live birth outcomes. However, clinical studies in humans are still very limited. Unresolved questions regarding technique and safety remain. For now, the approach remains experimental but promising [30].

### 2.4. Currently Available Conservative Therapies

Although MRT has shown promising safety and effectiveness, it remains prohibited in many countries, often without a strong scientific justification. As a result, couples seeking this treatment abroad face additional financial and emotional burdens. In such instances, it becomes important to consider clinically supported therapies that offer similar benefits without added challenges [18]. Age-related mitochondrial decline can compromise oocyte quality, prompting interest in therapies that support mitochondrial function [14]. Thus, antioxidants have been used during ARTs for improving outcomes [31]. Among these, coenzyme Q10 (CoQ10), melatonin, and resveratrol are the most extensively studied, with established benefits in conditions involving mitochondrial dysfunction, such as neurodegenerative and cardiovascular diseases [32].

#### 2.4.1. Mechanism and Rationale for CoQ10 Supplementation

CoQ10 functions as a major cellular antioxidant and a lipid-soluble electron carrier responsible for the transfer of electrons from complexes I and II to complex III in the mitochondrial respiratory chain. Investigators hypothesized the supplementation of CoQ10 two months before and during an IVF–ICSI cycle to improve oocyte energy production and reduce free oxygen radicals [32,33]. A randomized controlled trial involving 169 women with diminished ovarian reserve undergoing IVF/ICSI evaluated the effect of CoQ10 supplementation (600 mg/day for 60 days) before ovarian stimulation. A total of 76 women received 600 mg CoQ10 supplementation, and 93 women served as the control group. All participants belonged to POSEIDON Group 3 (women < 35 years with poor ovarian reserve), were diagnosed with primary infertility, and had not undergone any ART treatments. Statistical comparisons between groups were conducted using Student’s t-test or the Mann–Whitney U test for continuous variables and the chi-square or Fisher’s exact test for categorical variables, with statistical significance defined as *p* < 0.05. Both groups were comparable in baseline clinical characteristics. CoQ10 supplementation was well-tolerated throughout the study.

After 60 days of supplementation, day-3 FSH levels were significantly reduced compared to pre-treatment levels within the same group. In the treatment group, the clinical pregnancy rate and live birth rate per fresh embryo transfer cycle were 34.85% and 31.82% in women treated with CoQ10 and 25% and 21.88% in controls, respectively [32,33]. The CoQ10 group demonstrated a higher fertilization rate [67.49% vs. controls (*p* < 0.05)], reduced embryo transfer cancelation rate [8.33% vs. 22.89% (*p* = 0.04)], and higher cryopreservation rate [18.42% vs. 4.3% (*p* = 0.012)]. Pre-treatment with CoQ10 significantly reduced the total gonadotrophin dose required for ovarian response, shortened stimulation duration, elevated peak E2 levels, and improved fertilization rates, as well as the number of high-quality embryos. Although clinical pregnancy and live birth rates were higher in the CoQ10 group, these differences did not reach statistical significance in this study. Larger studies are needed to confirm effects on pregnancy and live birth rates, but CoQ10 is a promising conservative therapy for improving mitochondrial function in ART [34].

A more recent meta-analysis including six randomized controlled trials (n = 1529 participants) further supported these findings. CoQ10 pretreatment significantly improved several IVF outcomes like clinical pregnancy rate [OR = 1.84 (95% CI 1.33–2.53), *p* = 0.0002], number of oocytes retrieved [MD = 1.30 (95% CI 1.21–1.40), *p* < 0.00001], optimal embryo formation [OR = 0.59 (95% CI 0.21–0.96), *p* = 0.002], and cycle cancelation rate reduction [OR = 0.60 (95% CI 0.44–0.83), *p* = 0.002]. These results suggest that mitochondrial metabolic support can significantly influence oocyte competence and embryo development [25,31].

#### 2.4.2. Melatonin as a Conservative Mitochondrial Therapy in ART

Melatonin, an indoleamine hormone secreted by the pineal gland, is well known for regulating circadian rhythms, anti-aging mechanisms, and exerting potent antioxidant effects [35]. Many randomized controlled trials (RCTs) and multiple meta-analyses have consistently shown that melatonin supplementation may increase the proportion of MII oocytes, enhance endometrial thickness, increase clinical pregnancy rates, and reduce oxidative stress in follicular fluid. Variations in dosage and treatment protocols contribute to differences across studies. Melatonin, with its favorable safety profile, can be an excellent low-cost alternative for patients with diminished ovarian reserve or oxidative stress–related infertility [36].

#### 2.4.3. Clinical Implications of Resveratrol in Female Reproduction

Resveratrol is a natural polyphenolic compound (3-5-4′-trihydroxystilbene) with strong antioxidant properties. Previous research has documented that resveratrol may enhance mitochondrial biogenesis, imparting positive effects on the female reproductive system and encouraging potential therapeutic value. Women affected by polycystic ovary syndrome (PCOS) often exhibit delayed oocyte maturation, contributing to lower fertilization rates and impaired embryo implantation. Resveratrol supplements aim to inhibit follicular atresia, boost the reserve of ovarian follicles, and extend ovarian lifespan. A 60-day pretreatment period allows sufficient time for follicular maturation. Daily intake of up to 1000 mg of resveratrol has shown antioxidant effects in patients with PCOS or other health disorders [37]. Table 1 depicts the comparative overview of MRT and other related therapies.

### 2.5. Recent Advances in MRT Use in Patients with Subfertility Patients

Germline therapy is a form of gene therapy involving somatic and germline cells. Somatic cell therapies affect only the treated individual, whereas germline therapy modifies germline cells, meaning the changes affect both the individual and future offspring [15]. Mitochondria are crucial for regulating metabolism and supplying energy to embryos, directly impacting implantation potential. Dysfunction in oxidative phosphorylation genes can reduce mtRNA, altering oocyte quality. This has led to growing interest in assessing mtDNA copy number or relative mtDNA levels in human embryos as a potential biomarker for embryo viability [12].

Embryos were analyzed using standard molecular and genomic testing techniques. Researchers found that increased mtDNA in euploid embryos was linked to lower implantation potential due to metabolic deficits during oocyte maturation. The mitochondrial score (Ms) was defined as the ratio of mtDNA/nDNA to measure mitochondrial copy number per cell. Embryos with an Ms > 160 in blastomeres and >60 in blastocysts consistently failed to implant. In instances where pairs of transferred euploid embryos (one male, one female) led to single pregnancies, 6/10 implanted embryos exhibited lower relative mtDNA content. These findings underline the difficulty in establishing mtDNA copy number as a reliable biomarker of embryo viability. More prospective studies are needed to clarify results and improve measurement techniques [11].

### 2.6. Ethics and Regulations for Clinical Use of MRT

Mitochondrial replacement therapy raises several ethical concerns, particularly because it involves germline genetic modification that can be inherited by future generations. Critics argue that altering the germline without long-term safety data may expose descendants to unforeseen health risks and raises questions about consent from future individuals [38]. Ethical debates also focus on the concept of “three-parent babies,” as genetic material from a mitochondrial donor is introduced, challenging traditional ideas of parenthood and identity [39,40,41]. Additionally, concerns exist about potential misuse of the technology for non-therapeutic genetic enhancement, highlighting the need for strict regulatory oversight [42,43,44]. In the United States, research on MRT has been limited due to federal regulations. While standard IVF is allowed, MRT is not, because it involves a donor egg fragment in addition to the patient’s own genetic material. In 2014, the FDA reviewed MRT but concluded that there was insufficient animal data to move forward with human clinical trials. Later, the Institute of Medicine stated that MRT is ethically acceptable if used for women at high risk of passing on severe mitochondrial disease [16,45]. Globally, some countries have advanced further. The United Kingdom allows MRT under the Human Fertilization and Embryology Authority (HFEA), which oversees reproductive technologies. Clinical trials began after a 2015 amendment explicitly permitted mitochondrial replacement as part of IVF. Other countries, including Greece, Ukraine, Spain, Singapore, and Mexico, are exploring MRT, though many remain in early research phases. These differences highlight the ethical, legal, and practical challenges of making MRT widely available [15].

### 2.7. Case Study: Successful Use of MRT for Leigh Syndrome

A 36-year-old patient heteroplasmic for mtDNA mutation 8993T>G underwent MRT using vitrified spindles and donor cytoplasm. Of six reconstructed oocytes, two fertilized normally, and one developed to a blastocyst. The resulting child had a low mutation load ranging between 2.36 and 9.23% and remained healthy at 7 months of age, demonstrating MRT’s potential for preventing severe mitochondrial disease [46].

## 3. Methods

### 3.1. Search Strategy

For this review, we conducted a narrative literature search to explore the available evidence on the mitochondrial role in gametogenesis, fertilization, disease transmission, types of mitochondrial DNA modification, their uses, and legal and ethical implications. To ensure a comprehensive yet focused literature search, we used three major electronic databases—PubMed, Scopus, Web of Science, and Google Scholar search engine, as they offer extensive coverage of medical and clinical research. A structured search strategy was applied, using Medical Subject Headings (MeSH) terms and Boolean operators to identify relevant articles. The search targeted three primary themes: mitochondrial role in reproduction, available mitochondrial DNA replacement procedures, and aspects of its clinical application.

Keywords such as (“Mitochondrial DNA” OR mtDNA OR mitochondria* OR heteroplasmy), (“Reproductive Techniques, Assisted” OR assisted reproduction OR ART OR IVF OR ICSI), (mitochondrial replacement therapy OR mitochondrial donation OR spindle transfer OR pronuclear transfer OR ooplasmic transfer OR cytoplasmic transfer), and clinical applications were combined strategically to refine the results. The keywords were combined using Boolean operators to construct the final search string:

(“Mitochondrial DNA” OR mtDNA OR mitochondria*) AND (“Reproductive Techniques, Assisted” OR IVF OR ICSI OR ART) AND (mitochondrial replacement therapy OR mitochondrial donation OR spindle transfer OR pronuclear transfer OR ooplasmic transfer)

To ensure quality and relevance, we included peer-reviewed studies published in English, prioritizing original research and systematic reviews focusing on the themes. Studies that did not directly address mitochondrial modification or were not available in full-text English versions were excluded. After an initial search, we screened titles and abstracts to eliminate duplicates and non-relevant studies, and the full texts of selected articles were reviewed to assess their methodological rigor and contribution to the topic.

### 3.2. Data Collection

Four authors (MM, MA, FA, and HR) reviewed all titles independently. The potential relevance of the studies to be included for review was agreed on by discussion on a regular basis. Selected titles and abstracts were further screened between studies to reject the overlap of cases. Full-text copies of the selected papers were obtained, and the relevant data were extracted. The decision on exclusion or inclusion was made by discussion. The risk of bias was not assessed due to the nature of the studies. The images used in the manuscript are original. They are hand-drawn illustrations created by the authors, scanned, and the quality was enhanced by the CamScanner app (v7.12, INTSIG Information Co., Ltd., Singapore) [47].

## 4. Conclusions

Mitochondrial replacement therapy represents a significant advancement in reproductive medicine, offering hope to families affected by maternally inherited mitochondrial diseases. Techniques such as pronuclear transfer, spindle transfer, and polar body transfer, alongside supportive therapies like CoQ10, melatonin, and resveratrol, demonstrate the potential to improve reproductive outcomes while minimizing risks. Beyond the scientific promise, MRT carries profound ethical and social implications, reminding us that at the heart of these innovations are real people seeking a chance to have healthy, genetically related children. Responsible research, regulation, and application of these therapies are essential to ensure this hope can become a safe and accessible reality.

## Figures and Tables

**Table 1:** Comparative overview of MRT and related therapies.

Technique [Reference]	Method (Brief)	Main Outcome	Key Limitation
Mitochondrial Replacement Therapy [7]	Nuclear DNA transferred into a donor oocyte with healthy mitochondria	Prevents maternal mtDNA disease	Invasive; need for donor eggs; ethical/regulatory concerns
Stem Cell–Derived Mitochondrial Transfer [14]	Mitochondria from patients’ (anyone’s) stem cells (MSCs, oogonial stem cells)	Potential mitochondrial rejuvenation	Mostly preclinical; long-term safety unknown
AUGMENT [27]	Mitochondria from patient’s own germline (oogonial stem cells) injected into oocytes	Improves oocyte quality without donor mtDNA	Limited evidence; technically complex; OHSS risk
Mitoception [31]	Physical transfer of isolated mitochondria into cells	Enhances cellular mitochondrial function	Preclinical; integration and persistence unclear, unknown reproductive outcomes
Conservative Therapy (e.g., CoQ10) [32]	Pharmacologic support of existing mitochondria	Improves mitochondrial function indirectly	Supportive only; limited efficacy in severe disease

## Data Availability

No new data were created or analyzed in this study. Data sharing is not applicable to this article.

## Abbreviations

MRT	Mitochondrial Replacement Therapy
ATP	Adenosine Triphosphate
MtDNA	Mitochondrial DNA
nDNA	Nuclear DNA
ART	Assisted Reproductive Technologies
ICSI	Intracytoplasmic Sperm Injections
PNT	Pronuclear Transfer
ST	Spindle fiber transfer
PBT1	Polar Body 1 Transfer
PB2T	Polar Body 2 Transfer
MII	Metaphase II
GV	Germinal Vesicle
PGD	Preimplantation Genetic Diagnosis
HFEA	Human Fertilization and Embryology Authority
G RNA	Guide RNA
IVF	In Vitro Fertilization
RCTs	Randomized Controlled Trials
PCOS	Polycystic Ovary Syndrome
Ips	Induced Pluripotent Stem
GC	Granulosa Cells
